# Comparison of Cajal-like cells in pelvis and proximal ureter of kidney with and without hydronephrosis

**DOI:** 10.1590/S1677-5538.IBJU.2014.0427

**Published:** 2015

**Authors:** Ömer Balikci, Tahsin Turunç, Nebil Bal, Hüseyin Çelik, Hakan Özkardeş

**Affiliations:** 1 Department of Urology, Manisa Alaşehir State Hospital, Manisa, Turkey; 2 Department of Urology, School of Medicine, Başkent University, Adana, Turkey; 3 Department of Patology, School of Medicine, Başkent University, Adana, Turkey; 4 Department of Urology, School of Medicine, Inonu University, Malatya, Turkey

**Keywords:** Interstitial Cells of Cajal, Hydronephrosis, Ureter

## Abstract

**Objectives:**

To evaluate effects of Cajal-like cells on human renal pelvis and proximal ureter on peristalsis.

**Materials and Methods:**

63 patients submitted to nephrectomy due to atrophic non-functional kidney associated with hydroureteronephrosis were included as study group and 30 cases with nephrectomy due to other reasons were included as control group. Samples from renal pelvis and proximal ureters were obtained and sections of 5μ form paraffin blocks of these samples were prepared; layers of lamina propria and muscularis mucosa were examined by immune-histochemistry using CD117 in order to determine count and distribution of Cajal-like cells.

**Results:**

During immune-histochemical examinations of sections, obtained from renal pelvis and proximal ureter of hydronephrotic kidneys by CD117, Cajal-like cells number determined in lamina propria and muscularis propria was statistically significantly lower compared to control group (p<0.001). Distribution of Cajal-like cells in renal pelvis and proximal tubulus was similar under examination by light microscope, and also both groups were not different from each other regarding staining intensity of Cajal-like cells by c-kit.

**Conclusion:**

Significantly reduced number of Cajal-like cells in study group compared to control group, shows that these cells may have a key role in regulation of peristalsis at level of renal pelvis and proximal ureter in urinary system.

## INTRODUCTION

Cajal's interstitial cells have been identified by Ramon Cajal about 120 years ago as primitive intestinal neurons (1). Majority of studies about Cajal cells were conducted in gastrointestinal system. These cells were found to be localized in general within muscle layers and neighbored to nerve plexus. In several studies, Cajal cells were showed to be pacemaker cells controlling peristalsis in gastrointestinal system (GIS) (2). Reduction in number of these cells or anomalies in their distribution are considered to play a role in etiology of certain diseases associated with motility disorder of GIS (3).

Cajal-like cells (CLC) were demonstrated in urinary system firstly in 1999 (4). During animal studies, these cells were determined in rat vas deferens, rat ureter, guinea-pig prostate, guinea-pig urinary bladder and rabbit urethra. In humans, CLC were demonstrated in ureteropelvic junction (UPJ), renal pelvis, ureter, vesicouretral junction, urinary bladder and urethra (5–9). During their study, Solari and colleagues determined reduced number of these cells at congenital UPJ obstruction and stablish a role in the etiology of congenital UPJ obstruction (5). In another study, these cells were also suggested to be responsible for motility in rat vas deferens (6). Kuzgunbay et al. showed that CLC were found in rat ureter subjected to unilateral distal ureter obstruction, and these cells appeared to be increased during earlier phase of obstruction and then decreased during late phase. Consequently the conclusion was that these cells could be cells regulating motility of ureter (10).

In this study, count and distribution of CLC in renal pelvis and proximal ureter of patients who had undergone nephrectomy due to non-functional kidney associated with hydroureteronephrosis were compared to that of patients submitted to nephrectomy due to some other reasons and without hydroureteronephrosis. Our aim was to show whether CLC are reduced or not in renal pelvis and ureter of completely non-functional kidneys compared to control group. If CLC are significantly reduced in non-functional hydronephrotic kidneys, this would show that CLC are potentially among cells controlling peristalsis in pelvis and ureter.

## MATERIALS AND METHODS

Between January 2000 to February 2010, 63 patients with nephrectomy due to non-functional kidney associated with hydroureteronephrosis were included as study group and 30 patients with nephrectomy due to other reasons were included as control group at Başkent University Ankara central and Adana Teaching and Research Hospital. The patients without hydronephrosis whose collecting tubules system were intact with renal function and without systemic metastasis of renal tumors were chosen for control group. The study group was entirely composed of patients who developed hydroureteronephrosis due to ureteral obstruction and stone resulting in atrophic non-functional kidney. Out of the paraffin blocks of the samples obtained from kidney pelvis and proximal ureters, 5μ sections were prepared and the sections were put on slides with polylysine, and in order to determine count and distribution of CLC, their lamina propria and muscularis mucosa layers were examined immune-histochemically using CD117.

As mast cells are also positively stained by CD 117, these cells were discriminated from CLC by using toluidine blue before histopathological examination of CLC. Sections of 5μ were transferred on slides and they were kept within solution of toluidine blue (0.5 g toluidine and 100 mL distilled water, pH3) for an incubation period of 15 minutes following de-paraffinisation process, then they were rinsed by tap water, dried and kept within xylene for 5 minutes. Samples on slides were covered by lamella and examined under light microscope. Mast cells showed granular meta-chromatic staining.

Orange-brown staining was accepted as positive due to chromogen used in microscopic valuation of samples. Five serial sections were examined for each patient. Number of Cajal cells with CD117 positive was counted on lamina propria and muscularis propria of samples of renal pelvis and ureter at 10 HPF (x400) area and the mean was determined. Cells with round nucleus and cytoplasm with intensely granular staining were considered to be mast cells and cells with fusiform cytoplasm and nucleus were considered as CLC.

### Statistical analysis

Data analysis was done by SPSS for Windows 11.5. Shapiro Wilk test was used to determine whether distribution of continuous variables was close to normal or not. Descriptive analysis included: mean±standard deviation for age (minimum-maximum); median for cells count (minimum-maximum); and subject number and (%) for nominal variables. Student t test was used to investigate the significance between groups for mean age; and Mann Whitney test was used to investigate the significance of median cell count. Similarity of gender distribution was analyzed by Pearson χ-square test. Results were accepted statistically significant for p<0.05.

## RESULTS

The mean age was 43.5 (2–72 years) for 63 patients (31 women, 32 men) with nephrectomy due to atrophic hydronephrotic kidney. The mean age was 58.6 (38–82 years) for 30 patients (12 women, 18 men) with nephrectomy due to renal tumor and without hydronephrosis. At microscopic examination, CLC were brown due to chromogen used for immunohistochemical staining. These cells stained by anti c-kit showed radial branched extensions. The distribution of CLC number according to groups is shown on Table-1.

**Table 1:** Number of Cajal-like cells on lamina propria and muscularis propria of renal pelvis and proximal ureter according to groups.

	Cajal-like cell numbers		
	Control Group 32 (26–42)	Study Group 22 (14–28)	P
Renal pelvis lamina propria	32 (26–42)	22 (14–28)	<0.001
Renal pelvis muscularis propria	42 (34–64)	26 (15–36)	<0.001
Proximal ureter lamina propria	24 (20–26)	12 (9–20)	<0.001
Proximal ureter muscularis propria	29 (25–32)	17 (10–23)	<0.001

Data are shown as median (minimum-maximum), Mann Whitney U test.

**Figure 1:**
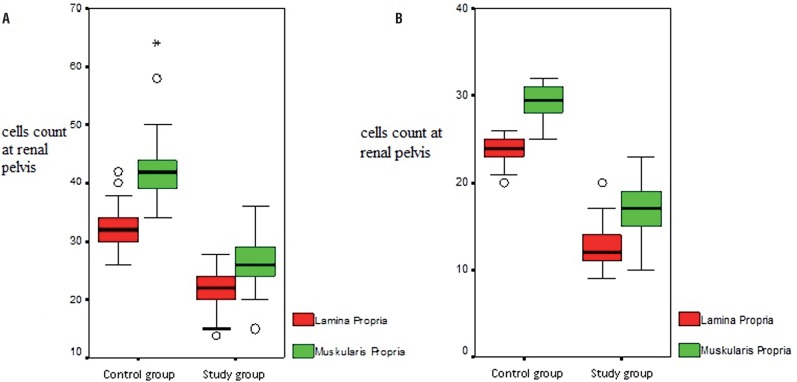
A) Cajal-like cells count at renal pelvis lamina propria and muscularis propria according to groups. B) Cajal's cell count at proximal ureter lamina propria and muscularis propria according to groups.

Cajal-like cells count determined on lamina propria and muscularis propria obtained from sections of hydronephrotic renal pelvis by immunohistochemical CD117 examination was statistically significantly reduced compared to control group (Figure-1A). Distribution of CLC in proximal ureter and renal pelvis of both study and control groups were similar under light microscopy and also staining intensity of CLC by c-kit was not different between both groups.

**Figure 2:**
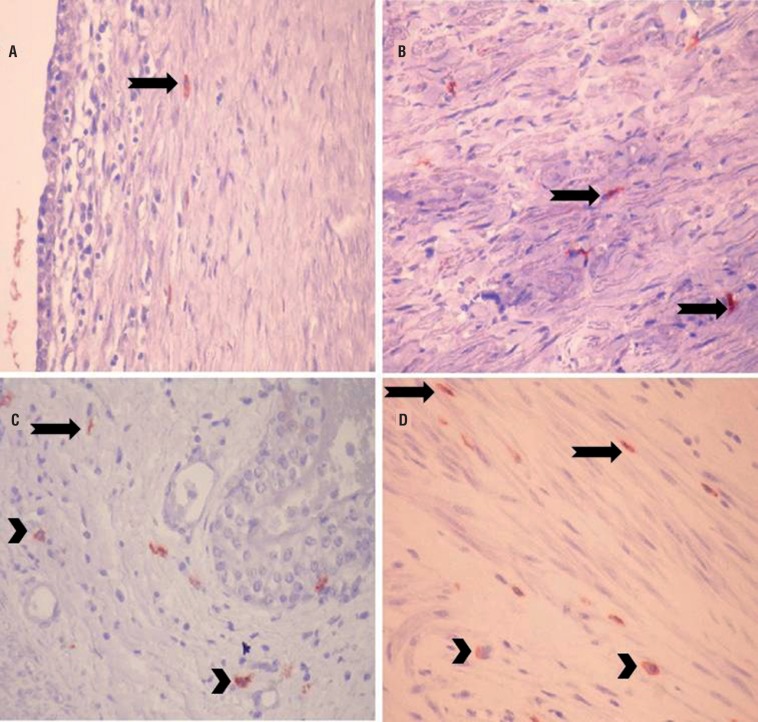
Cajal-like cells on renal pelvis lamina propria layer of study group (a and b) and control group (c and d) (CD117; x400) (Arrows: Cajal cells, arrowheads: mast cells).

Cajal-like cells determined on renal pelvis lamina propria and muscularis propria in study group and control group are shown in Figure-2.

**Figure 3:**
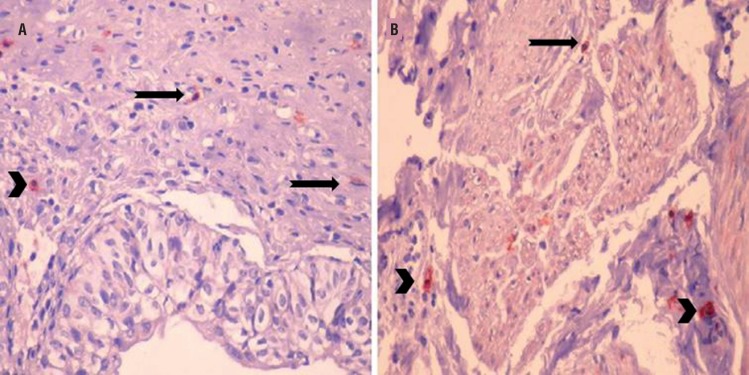
Cajal-like cells on proximal ureter lamina propria layer of study group (a and b) and mast cells (a) (CD117; x400) (Arrows: Cajal cells, arrowheads: mast cells).

Cajal-like cells count determined on both lamina propria and muscularis propria of sections obtained from proximal ureter was statistically significantly reduced compared to control group (Figure-1B). Cajal-like cells determined on proximal ureter lamina propria and muscularis propria in study group are shown in Figure-3.

## DISCUSSION

In this study based on results showing that CLC play a role in regulation of urinary system motility as in gastrointestinal system, significant reduction in number of Cajal cells with CD117 positive staining was shown in layers of lamina propria and muscularis propria of renal pelvis and proximal ureter of patients submitted to nephrectomy due to hydroureteronephrosis. This result indicates that interstitial CLC are also closely associated with peristalsis in urinary system.

Cajal-like cells have been firstly identified as primitive intestinal neurons in 1893, and then they have been associated with neurons and intestinal smooth muscle cells (1). Several studies showed that these cells are pacemaker cells localized between nerve ending and smooth muscle cells in GIS and responsible of transmitting slow electrical waves necessary for peristaltic movement (2). Additionally, reduced c-kit immune-reactivity and difference in CLC distribution were observed during certain gastrointestinal disorders associated with impaired peristaltic movement such as Hirschsprung's disease, infantile hypertrophic pyloric stenosis, slow-passage constipation (11–13).

In the studies carried out during following years, interstitial CLC were detected in all organs of the urinary system and connection between them and urinary system disorders were tried to be revealed (5–10). Studies on CLC in urinary system suggest that these cells have pacemaker activity especially in tubular organs and that they may induce peristalsis. Although interstitial CLC have been shown in urinary system in the last 13 years, number of studies on these cells in both human and experimental animals is recently increasing. Majority of cases included in these studies are patients with anatomical or functional obstruction on various regions of urinary system or with experimentally induced obstruction. Demonstration of statistically reduced number of these cells at proximal of obstruction and at obstructed segment in gastrointestinal system, especially in cases with intestinal obstruction, compared to control groups, suggests that CLC could also have similar properties within urinary system.

In a study carried out in recent years in order to understand vesicoureteral reflux mechanisms and structural changes they induce in the urinary system, 32 patients with vesicoureteral reflux at varying degrees were compared with 8 control cases regarding presence of intramural c-kit positivity cell and it was determined that collagen stroma replaced smooth muscle bundles and these was remarkable reduced in interstitial cells. It was accepted that interstitial CLC provided automatic rhythm and ureteral peristaltism coordination in ureters and a relation was thought to exist between the loss of interstitial CLC and vesicoureteral reflux and impaired active ureteral valve mechanisms (14). Another study conducted in patients with primary obstructive mega-ureter determined that distribution of interstitial cells stained positively by c-kit was normal in longitudinal and circular muscle layers at dilated segments localized on proximal part of obstruction, but it showed also that there was remarkable smooth muscle hypoplasia in obstructed segment and number of interstitial cells was reduced or no interstitial cells were present. Absence of CLC in longitudinal muscle layer is explained by the absence of c-kit positive embryological muscle cells precursors (15).

Solari and colleagues determined that human UPJ contained many c-kit positive Cajal cells and their number was very low in obstruction of ureteropelvic junction compared to control group or they were completely absent in UPJ obstruction. In that study, it was claimed that CLC were responsible of ureter peristalsis (5). Kuzgunbay et al. created experimental obstruction in distal ureter of a total of 175 rats and they investigated changes in number and morphology of CLC in UPJ after obstruction. A statistically significant increase was determined in mean number of CLC in study group compared to control group and that these cells decreased during late phase. Authors suggested that the cause of increased number of CLC following first days of obstruction was the differentiation of CLC precursors in response to increased peristaltic activity during early phase. However, the cause of discontinuation of this increase in spite of reduced peristalsis during late phase was associated with prolonged neuromodulator role of CLC in chronic obstruction (10). In our study, statistically significant reduction in number of interstitial CLC stained positively by CD117 was determined at renal pelvis and proximal ureter in patients with nephrectomy due to hydronephrosis compared to control group. In the study of Kuzgunbay and colleagues, CLC number was evaluated after maximal 90 days following obstruction. In our study, changes in CLC number that occurred during years following obstruction were investigated. In the light of the data from these both studies, the study of Kuzgunbay and colleagues may be considered as acute and sub-acute period and our study may be considered as chronic period. It may be concluded that there is an increase of CLC number associated with increased peristaltic activity due to obstruction during early period and also reduction of CLC number associated with loss of peristalsis during chronic period.

Kuvel and colleagues conducted a study to investigate underlying basic histopathology at intrinsic UPJ obstruction and to associate this with surgical treatment results, and they examined the obstructed segment of 32 cases with intrinsic UPJ obstruction; the segment of UPJ with chronic obstruction in 15 cases with nephrectomy due to chronic obstruction associated with lithiasis, tumor and reflux; and lastly they examined also normal ureteropelvic junction segment of 30 patients submitted to nephrectomy due to renal tumor or trauma; then they evaluated CLC in these segments. Cells positive with c-kit for immunohistochemical staining of CD117 monoclonal antibody were generally spread and in rare lining and they were not in regular interaction. These cells were observed to have morphological aspects of CLC cited in literature and were readily discriminated from mast cells showing positive staining. When distribution and density of C-kit positive Cajal cells alterations at intrinsic UPJ obstruction segments were compared to normal ureter segments and chronically obstructed ureter segments, intrinsic UPJ obstruction segments were similar except proximally to surgical area limit and no statistically significant difference was present. However, c-kit positive cells were higher at proximal and distal edge margins of intrinsic UPJ segments compared to obstruction area. According to these results, authors suggested that CLC are responsible of intermediate mechanisms having a role between pacemaker cells and innervations in state of a direct role in pathogenesis of intrinsic UPJ obstructions (16).

When we go over the studies that Solari and Kuvel related to CLC in UPJ junction obstruction, the fact that these cells decrease in cases with UPJ obstruction compared to control group stands out. This result may be interpreted as these cells don't function in functionally or anatomically obstructed UPJ. In our study, proximal ureter and renal pelvic segments without any peristaltic function were evaluated and our findings and results of studies conducted in patients with UPJ obstruction are similar, since in our study number of CLC in muscularis propria and lamina propria of non-functional pelvis and proximal ureter are significantly lower. Therefore according to these results, CLC could be reduced in ureter with loss of motility and their function could also be diminished.

In recent years, studies about localization, number, function of CLC in urinary system and their role in pathophysiology of urinary system disorders are increasing in parallel to advances in pathological and immunohistochemical diagnostic tools. In electron microscopy trials, morphological alterations of CLC in obstructed urinary system may be revealed and if necessary, in association with electrophysiological trials, most of unknown ultrastructural changes of these cells may be demonstrated.

## CONCLUSIONS

Neurophysiological trials in association with better understanding of association of structural aspects and peristalsis of CLC in urinary system, will lead to better understanding of function of these cells in urinary system and the development of new treatment modalities for urinary system pathologies related with peristalsis.
